# Five-year cardiovascular outcomes across hypertensive disorders of pregnancy subtypes: a propensity-matched analysis

**DOI:** 10.3389/fcvm.2026.1875409

**Published:** 2026-07-24

**Authors:** Laith Alhuneafat, Omar Obeidat, Dina Mohammed, Nikita Sumzin, Kamel Ikbariah, Ahmad Al-Abdouh, Ahmad Jabri, Bhavadharini Ramu, Jessica Schultz, Jessica Gottula, Bethany A. Sabol, Selma Carlson

**Affiliations:** 1 Division of Cardiovascular Medicine, University of Minnesota, Minneapolis, MN, United States; 2 Division of Cardiovascular Medicine, University of Texas Medical Branch, Galveston, TX, United States; 3 School of Medicine, University of Jordan, Amman, Jordan; 4 Department of Medicine, Lahey Hospital & Medical Center, Burlington, MA, United States; 5 Department of Cardiology, Beaumont Health, Royal Oak, MI, United States; 6 Department of Maternal-Fetal Medicine, University of Minnesota, Minneapolis, MN, United States

**Keywords:** chronic hypertension, gestational hypertension (GH), hypertensive disorders of pregnancy (HDP), postpartum cardiovascular risk, preeclampsia, superimposed preeclampsia

## Abstract

**Background:**

Hypertensive disorders of pregnancy (HDP) are established risk factors for future cardiovascular disease, yet long-term cardiovascular risk across specific HDP subtypes remains incompletely defined.

**Methods:**

We conducted a retrospective cohort study using a large, global electronic health record network to evaluate five-year cardiovascular outcomes among women with gestational hypertension, chronic hypertension, preeclampsia/eclampsia, and superimposed preeclampsia compared with normotensive pregnancies. Propensity score matching was performed to balance baseline demographic and cardiometabolic risk factors. Outcomes included heart failure, ischemic heart disease, stroke, pulmonary embolism, atrial fibrillation or flutter, and major adverse cardiovascular events (MACE).

**Results:**

After propensity score matching, 109,945 women with gestational hypertension, 36,560 with chronic hypertension, 94,198 with superimposed preeclampsia, and 80,848 with preeclampsia/eclampsia were each matched 1:1 with normotensive controls. Superimposed preeclampsia was associated with the largest observed odds of adverse cardiovascular outcomes, including heart failure (OR 3.80, 95% CI 3.46–4.16), stroke (OR 3.66, 95% CI 3.35–4.00), ischemic heart disease (OR 3.08, 95% CI 2.83–3.36), and MACE (OR 3.29, 95% CI 3.11–3.47). Chronic hypertension and preeclampsia/eclampsia demonstrated comparatively elevated risk, with chronic hypertension associated with increased odds of heart failure (OR 2.88, 95% CI 2.43–3.43) and MACE (OR 2.24, 95% CI 2.01–2.49), and preeclampsia/eclampsia associated with higher odds of heart failure (OR 3.10, 95% CI 2.62–3.65) and stroke (OR 2.29, 95% CI 1.99–2.63). Gestational hypertension was associated with increased odds of heart failure (OR 1.35, 95% CI 1.14–1.60) but not with most other cardiovascular outcomes.

**Conclusions:**

Long-term cardiovascular risk following HDP varies by subtype, with all major subtypes demonstrating increased risk compared with normotensive pregnancies and larger effect estimates observed among women with superimposed preeclampsia. These findings support consideration of hypertensive disorder subtype in postpartum cardiovascular risk assessment.

## 1 Introduction

Hypertensive disorders of pregnancy (HDP) are common and increasingly diagnosed pregnancy-related complications in the United States (1, 2). The HDP spectrum includes chronic hypertension, gestational hypertension, preeclampsia/eclampsia, and chronic hypertension with superimposed preeclampsia, with subtype definitions determined largely by timing of hypertension onset and the presence of end-organ features. Among U.S. delivery hospitalizations in the National Inpatient Sample, HDP prevalence increased from 12.0% to 18.8% from 2015 through 2021 which highlights the growing public health relevance of these conditions (2). Beyond immediate obstetric consequences, HDPs are established markers of increased later-life cardiovascular disease (CVD) risk, consistent with pregnancy being a physiologic cardiovascular “stress test” that may unmask predisposition to cardiometabolic disease (3–7).

Multiple studies and meta-analyses have shown that history of HDP is associated with higher risks of subsequent heart failure, ischemic heart disease, stroke, and other cardiovascular complications (5, 8). Earlier studies often grouped HDP subtypes together, focused primarily on preeclampsia, or evaluated a limited range of cardiovascular outcomes. More recent investigations have advanced the field by examining subtype-specific risk (3, 9). Hu et al. (10) evaluated various HDP subtypes, gestational age at onset, and seven cardiovascular outcomes within five years after delivery in a large Florida cohort, while Kwak et al. (11) demonstrated distinct long-term cardiovascular risk patterns across various HDP subtypes in a nationwide South Korean cohort. These studies highlight the clinical importance of distinguishing among HDP phenotypes, but their geographically defined populations and differing analytic approaches leave uncertainty regarding the reproducibility of these patterns across broader healthcare settings.

HDP subtypes share overlapping pathophysiologic pathways but differ in the timing and chronicity of hypertension and in the presence of pregnancy-related end-organ involvement, distinctions that may translate into different cardiovascular risk profiles (12, 13). The present study builds on prior work by evaluating four major HDP subtypes within a large, global, federated electronic health record network using a uniform hierarchical classification, separate propensity score-matched normotensive control cohorts, a common five-year postpartum window, and the same cardiovascular endpoints. This design allows direct comparison of early postpartum cardiovascular risk across HDP phenotypes and provides a clinically interpretable assessment of whether previously reported subtype-specific patterns are reproducible across heterogeneous healthcare systems (8, 10, 14).

## 2 Methodology

### 2.1 Data source

Data for this study were obtained from the TriNetX Research Network, a global federated database that compiles de-identified electronic health records from over 106 healthcare organizations. The database includes comprehensive patient-level information such as demographics, diagnoses, procedures, medications, and laboratory results. All analyses were performed within the TriNetX environment, ensuring compliance with HIPAA and patient privacy standards.

### 2.2 Study design and population

This was a retrospective cohort study evaluating long-term cardiovascular outcomes among women with HDP compared to those with normotensive pregnancies. Eligible participants were females aged 18 to 55 years who had a delivery encounter between January 01, 2010, and December 01, 2025, identified through ICD-9 and ICD-10 codes for delivery and related procedures. Ectopic pregnancy, hydatidiform mole, spontaneous abortion, induced-termination complications, failed attempted termination, complications following ectopic or molar pregnancy, and abortion procedures were excluded. Preterm birth, fetal growth restriction, and other non-hypertensive obstetric complications were not exclusion criteria and therefore could be present in either the HDP or control cohorts. The complete eligibility and exclusion code set is provided in Supplementary Table S1.

### 2.3 Exposure classification

To avoid duplication and ensure independence of observations, each individual was included only once using the first qualifying delivery encounter (index pregnancy). Hypertensive disorder subtypes were defined based on diagnoses temporally linked to this index pregnancy, and diagnoses occurring outside the index pregnancy were not used for exposure classification. The study population was categorized into four mutually exclusive hypertensive subgroups using a hierarchical classification approach based on ICD-9 and ICD-10 diagnostic codes. The hierarchy was selected to reflect clinical diagnostic precedence and to prevent a pregnancy with overlapping codes from contributing to more than one exposure cohort, consistent with guideline definitions and previously published rule-based and hierarchical subtype approaches (10–13, 15). To ensure that each patient was assigned to a single group, categories were defined and applied sequentially. First, patients meeting criteria for superimposed preeclampsia were identified, defined as those with pre-existing hypertension and concurrent preeclampsia/eclampsia (ICD-10: O11; ICD-9: 642.7), or the presence of both chronic hypertension (ICD-10: O10, I10–I15) and preeclampsia/eclampsia codes (ICD-10: O14, O14.2, O15). These patients were classified exclusively as superimposed preeclampsia.

Among the remaining patients, those with preeclampsia, eclampsia, or HELLP syndrome (ICD-10: O14, O14.2, O15; ICD-9: 642.4) without evidence of chronic hypertension were classified as preeclampsia/eclampsia. Next, patients with chronic hypertension complicating pregnancy (ICD-10: O10, I10–I15) without superimposed preeclampsia were classified as chronic hypertension. Finally, patients with gestational hypertension without significant proteinuria (ICD-10: O13), excluding those with preeclampsia or chronic hypertension, were classified as gestational hypertension.

This approach is conceptually aligned with the mutually exclusive hierarchical algorithm used by Kwak et al. (11) and the rule-based phenotyping strategy used by Hu et al. (10), but was adapted to the data structure and objective of the present study. Unlike Hu et al., whose linked dataset permitted incorporation of blood pressure, laboratory, medication, and gestational-age information, our algorithm used diagnostic codes that could be applied consistently across participating TriNetX healthcare organizations. Unlike Kwak et al., we did not analyze unspecified hypertension as a fifth exposure because the study objective was to evaluate four clinically defined HDP subtypes. The control group comprised pregnancies meeting the same age, delivery, and pregnancy-loss exclusion criteria as the HDP cohorts but with no diagnosis of HDP or related hypertensive pregnancy complications. All HDP-associated ICD-9/10 codes, including unspecified categories, were excluded from the control cohort; preterm birth, fetal growth restriction, and other non-hypertensive obstetric complications were not excluded (Supplementary Table S1).

### 2.4 Index event and follow-up

The index event was defined as the date of delivery. Follow-up began one day after the index event and continued for 1,825 days (five years).

### 2.5 Outcomes

Primary outcomes included incident cardiovascular events occurring within five years after delivery. These outcomes included heart failure, atrial fibrillation, stroke, pulmonary embolism, and major adverse cardiovascular events (MACE), defined as a composite of stroke and other cerebrovascular events, ischemic heart disease, heart failure, and all-cause mortality. All outcomes were identified using validated ICD-9 and ICD-10 diagnostic codes available within the TriNetX database.

### 2.6 Propensity score matching

Each hypertensive subgroup was compared to a matched control group using 1:1 propensity score matching. Logistic regression models were used to calculate propensity scores based on baseline demographic and clinical variables including age, race, diabetes, obesity, dyslipidemia, smoking, chronic kidney disease, and previous cardiovascular disease. Matching was conducted using a greedy nearest-neighbor algorithm with a caliper width of 0.1 pooled standard deviations to minimize imbalance between cohorts. Covariate balance was assessed using standardized mean differences, with values <0.1 considered indicative of adequate balance.

### 2.7 Statistical analysis

Associations between HDP and cardiovascular outcomes were expressed as odds ratios with 95% confidence intervals. A *p*-value <0.05 was considered statistically significant. All analyses were performed within the TriNetX analytics environment using its built-in statistical tools.

### 2.8 *Post hoc* analysis

In a *post hoc* analysis, we evaluated a composite postpartum hypertension outcome during the five-year follow-up period. The outcome was defined as a new diagnosis of essential (primary) hypertension or documentation of an antihypertensive medication from the following classes: beta blockers, alpha blockers, calcium-channel blockers, antihypertensive combinations, other antihypertensives, peripheral vasodilators, angiotensin-converting enzyme inhibitors, angiotensin II receptor inhibitors, direct renin inhibitors, thiazide or related diuretics, potassium-sparing or combination diuretics, and other diuretics. Patients meeting this composite outcome before the follow-up window were excluded from the analysis. The analysis was performed for gestational hypertension, preeclampsia/eclampsia, and severe preeclampsia/eclampsia and their respective matched controls.

### 2.9 Sensitivity analysis

To assess whether the cardiovascular risk associated with pre-eclampsia/eclampsia was driven by more severe disease phenotypes, we conducted a pre-specified sensitivity analysis restricted to women with preeclampsia with severe features, eclampsia, or HELLP syndrome (ICD-10: O14.1, O14.2, O15; ICD-9: 642.5). This excluded women with preeclampsia without severe features or unspecified preeclampsia (ICD-9: 642.4), gestational hypertension (ICD-10: O13; ICD-9: 642.3), pre-existing chronic hypertension (ICD-10: O10; ICD-9: 642.2), and superimposed pre-eclampsia (ICD-10: O11; ICD-9: 642.7). The exposure cohort was matched 1:1 to a normotensive control cohort using the same propensity score model and matching algorithm described in Section 2.6. The follow-up window and statistical approach were identical to the primary analysis, with associations expressed as odds ratios with 95% confidence intervals.

## 3 Results

### 3.1 Baseline characteristics

Following propensity score matching, four HDP cohorts were each matched 1:1 with normotensive controls. The final analytic sample included 109,945 women with gestational hypertension, 36,560 with chronic hypertension, 94,198 with superimposed preeclampsia, and 80,848 with preeclampsia/eclampsia, with equal numbers of matched controls. Mean maternal age varied modestly across subtypes, with numerically higher values in superimposed preeclampsia (32.3 ± 6.4 years) and chronic hypertension (31.0 ± 6.0 years), and lower in gestational hypertension (29.2 ± 6.0 years) and preeclampsia/eclampsia (29.1 ± 6.4 years), with good balance after matching.

Racial and ethnic composition differed across HDP subtypes. White patients represented the largest group across cohorts (61.5% in gestational hypertension and ∼51% in other subtypes). The proportion of Black patients varied across subtypes, with higher representation in superimposed preeclampsia (30.4%) compared with gestational hypertension (18.6%). Hispanic ethnicity was most prevalent in the preeclampsia/eclampsia cohort (21.2%).

Cardiometabolic comorbidities showed variation across subtypes, with higher prevalence observed in certain groups. Obesity and diabetes were most prevalent in superimposed preeclampsia (37.6% and 9.2%, respectively), with elevated prevalence also observed in chronic hypertension, and were less frequent in gestational hypertension and preeclampsia/eclampsia. Dyslipidemia, chronic kidney disease, and nicotine dependence were also more common in superimposed preeclampsia and chronic hypertension. Asthma was prevalent across all cohorts, with the highest rates in superimposed preeclampsia (15.4%).

Pre-existing cardiovascular disease was uncommon overall. Heart failure prevalence was numerically higher in superimposed preeclampsia (1.1%) and chronic hypertension (0.8%), and lower in gestational hypertension (0.1%) and preeclampsia/eclampsia (0.3%), while atrial fibrillation and ischemic heart disease were rare across all groups. Baseline characteristics were well balanced between hypertensive and matched control cohorts (Supplementary Tables S2–S5).

### 3.2 Clinical outcomes

**Figure 1:**
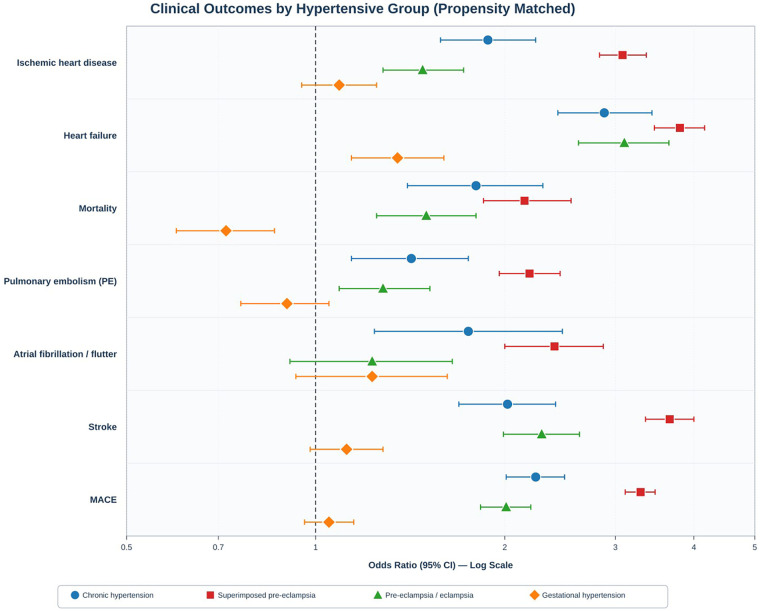
Association between hypertensive disorders of pregnancy and long-term cardiovascular outcomes. Forest plot showing adjusted odds ratios (ORs) and 95% confidence intervals (CIs) for cardiovascular outcomes within five years postpartum among women with gestational hypertension, chronic hypertension, preeclampsia/eclampsia, and superimposed preeclampsia compared with propensity score–matched normotensive controls. Outcomes include ischemic heart disease, heart failure, all-cause mortality, pulmonary embolism, atrial fibrillation or flutter, stroke, and major adverse cardiovascular events (MACE). Each hypertensive disorder subtype was matched to an independent control cohort, and estimates are presented relative to the respective matched control group. The vertical dashed line indicates the null value (OR = 1.0). OR, odds ratio; CI, confidence interval; MACE, major adverse cardiovascular events.

Adjusted odds ratios for clinical outcomes are presented in Table 1. Compared with normotensive controls, hypertensive disorder subtypes demonstrated a pattern of increasing cardiovascular risk across subtypes when comparing effect estimates, with the largest effect estimates observed in superimposed preeclampsia, while chronic hypertension and preeclampsia/eclampsia also showed elevated odds, and gestational hypertension showed more limited associations (Figure 1).

**Table 1:** Event rates and adjusted odds ratios for clinical outcomes after propensity score matching.

Outcome	Gestational HTN *n* (%) vs. control	OR (95% CI)	Pre-eclampsia/Eclampsia *n* (%) vs. control	OR (95% CI)	Chronic HTN *n* (%) vs. control	OR (95% CI)	Superimposed Pre-eclampsia *n* (%) vs. control	OR (95% CI)
Ischemic heart disease	422 (0.4%) vs. 389 (0.4%)	1.09 (0.95–1.25)	425 (0.5%) vs. 287 (0.4%)	1.48 (1.28–1.72)	365 (1.0%) vs. 195 (0.5%)	1.88 (1.58–2.24)	2,168 (2.3%) vs. 714 (0.8%)	3.08 (2.83–3.36)
Heart failure	307 (0.3%) vs. 228 (0.2%)	1.35 (1.14–1.60)	576 (0.7%) vs. 187 (0.2%)	3.10 (2.62–3.65)	503 (1.4%) vs. 176 (0.5%)	2.88 (2.43–3.43)	2,182 (2.3%) vs. 585 (0.6%)	3.80 (3.46–4.16)
Pulmonary embolism (PE)	281 (0.3%) vs. 314 (0.3%)	0.90 (0.76–1.05)	308 (0.4%) vs. 240 (0.3%)	1.28 (1.09–1.52)	202 (0.6%) vs. 143 (0.4%)	1.42 (1.14–1.75)	985 (1.0%) vs. 452 (0.5%)	2.19 (1.96–2.45)
Atrial fibrillation/flutter^a^	113 (0.1%) vs. 92 (0.1%)	1.23 (0.93–1.62)	97 (0.1%) vs. 79 (0.1%)	1.23 (0.91–1.65)	89 (0.2%) vs. 51 (0.1%)	1.75 (1.24–2.47)	406 (0.4%) vs. 170 (0.2%)	2.40 (2.00–2.87)
Stroke	440 (0.4%) vs. 394 (0.4%)	1.12 (0.98–1.28)	663 (0.8%) vs. 291 (0.4%)	2.29 (1.99–2.63)	363 (1.0%) vs. 181 (0.5%)	2.02 (1.69–2.41)	2,285 (2.4%) vs. 636 (0.7%)	3.66 (3.35–4.00)
MACE	1,037 (0.9%) vs. 988 (0.9%)	1.05 (0.96–1.15)	1,423 (1.8%) vs. 716 (0.9%)	2.01 (1.83–2.20)	1,069 (2.9%) vs. 486 (1.3%)	2.24 (2.01–2.49)	5,329 (5.7%) vs. 1,688 (1.8%)	3.29 (3.11–3.47)
New onset hypertension or antihypertensive treatment^b^	20,560/109,945 (18.7%) vs. 9,565/109,945 (8.7%)	2.41 (2.33–2.49)	25,386/80,848 (31.4%) vs. 6,468/80,848 (8.0%)	5.29 (5.09–5.49)	Not applicable	-	Not applicable	

Values shown as *n* (%) vs. matched control. Odds ratios (OR) with 95% confidence intervals (CI).

a Atrial fibrillation/flutter analyses exclude patients with the outcome prior to the time window.

b Post-hoc analysis.

Superimposed preeclampsia was associated with the largest effect estimates across all outcomes, including heart failure (OR 3.80, 95% CI 3.46–4.16), stroke (OR 3.66, 95% CI 3.35–4.00), ischemic heart disease (OR 3.08, 95% CI 2.83–3.36), atrial fibrillation or flutter (OR 2.40, 95% CI 2.00–2.87), pulmonary embolism (OR 2.19, 95% CI 1.96–2.45), and MACE (OR 3.29, 95% CI 3.11–3.47).

Chronic hypertension demonstrated consistently elevated risk across outcomes, with increased odds across all outcomes, including heart failure (OR 2.88, 95% CI 2.43–3.43), stroke (OR 2.02, 95% CI 1.69–2.41), ischemic heart disease (OR 1.88, 95% CI 1.58–2.24), atrial fibrillation or flutter (OR 1.75, 95% CI 1.24–2.47), pulmonary embolism (OR 1.42, 95% CI 1.14–1.75), and MACE (OR 2.24, 95% CI 2.01–2.49).

Similarly, preeclampsia/eclampsia was associated with increased odds of heart failure (OR 3.10, 95% CI 2.62–3.65), stroke (OR 2.29, 95% CI 1.99–2.63), ischemic heart disease (OR 1.48, 95% CI 1.28–1.72), pulmonary embolism (OR 1.28, 95% CI 1.09–1.52), and MACE (OR 2.01, 95% CI 1.83–2.20), while associations with atrial fibrillation or flutter were not statistically significant.

In contrast, gestational hypertension was associated with a modest increase in heart failure risk (OR 1.35, 95% CI 1.14–1.60), without significant associations for other cardiovascular outcomes.

### 3.3 *Post hoc* analysis: New-onset hypertension

Within five years, the composite outcome occurred in 20,560 of 109,945 women with gestational hypertension (18.7%) compared with 9,565 of 109,945 matched controls (8.7%), corresponding to more than a twofold higher risk (OR 2.41, 95% CI 2.33–2.49; HR 2.65, 95% CI 2.57–2.73). Among women with preeclampsia/eclampsia, the composite outcome occurred in 25,386 of 80,848 women (31.4%) compared with 6,468 of 80,848 matched controls (8.0%), reflecting an approximately fivefold higher risk (OR 5.29, 95% CI 5.09–5.49; HR 4.89, 95% CI 4.73–5.06).

### 3.4 Sensitivity analysis: severe pre-eclampsia/eclampsia

After 1:1 propensity matching, 7,701 women with severe pre-eclampsia, eclampsia, or HELLP syndrome were compared with 7,701 normotensive controls. Baseline characteristics were well balanced after matching, with all standardized differences below 0.02 (Supplementary Table S6). Compared with normotensive controls, severe pre-eclampsia/eclampsia was associated with significantly increased odds of heart failure (OR 4.04, 95% CI 2.53–6.44), stroke (OR 3.20, 95% CI 2.20–4.66), MACE (OR 2.55, 95% CI 1.96–3.31), pulmonary embolism (OR 1.84, 95% CI 1.12–3.03), and ischemic heart disease (OR 1.68, 95% CI 1.05–2.69) within five years of delivery (Supplementary Table S7). In the *post hoc* analysis, the new-onset hypertension occurred in 3,301 of 7,701 women (42.9%) compared with 613 of 7,701 matched controls (8.0%), corresponding to an almost ninefold higher odds of the outcome (OR 8.68, 95% CI 7.62–9.89).

## 4 Discussion

In this large, propensity score–matched analysis of women with HDP, we observed a pattern of subtype-specific associations, with cardiovascular outcomes within five years after delivery differing across HDP subtypes. Compared with normotensive pregnancies, chronic hypertension, preeclampsia/eclampsia, and superimposed preeclampsia were each associated with significantly higher odds of multiple adverse cardiovascular outcomes, including heart failure, ischemic heart disease, stroke, pulmonary embolism, and major adverse cardiovascular events (Central figure). The magnitude of association was substantial, with approximately three- to four-fold higher odds for key outcomes in superimposed preeclampsia, and moderate elevations in risk for chronic hypertension and preeclampsia/eclampsia. Notably, superimposed preeclampsia and chronic hypertension were also associated with atrial fibrillation or flutter, while preeclampsia/eclampsia was not. In contrast, gestational hypertension was associated with a more limited cardiovascular risk profile, showing increased odds of heart failure but no significant associations with most other cardiovascular outcomes. These findings are consistent with (and extend) emerging evidence that HDP is not a monolith and that subtype carries clinically meaningful prognostic information (10, 11).

**CENTRAL FIGURE:**
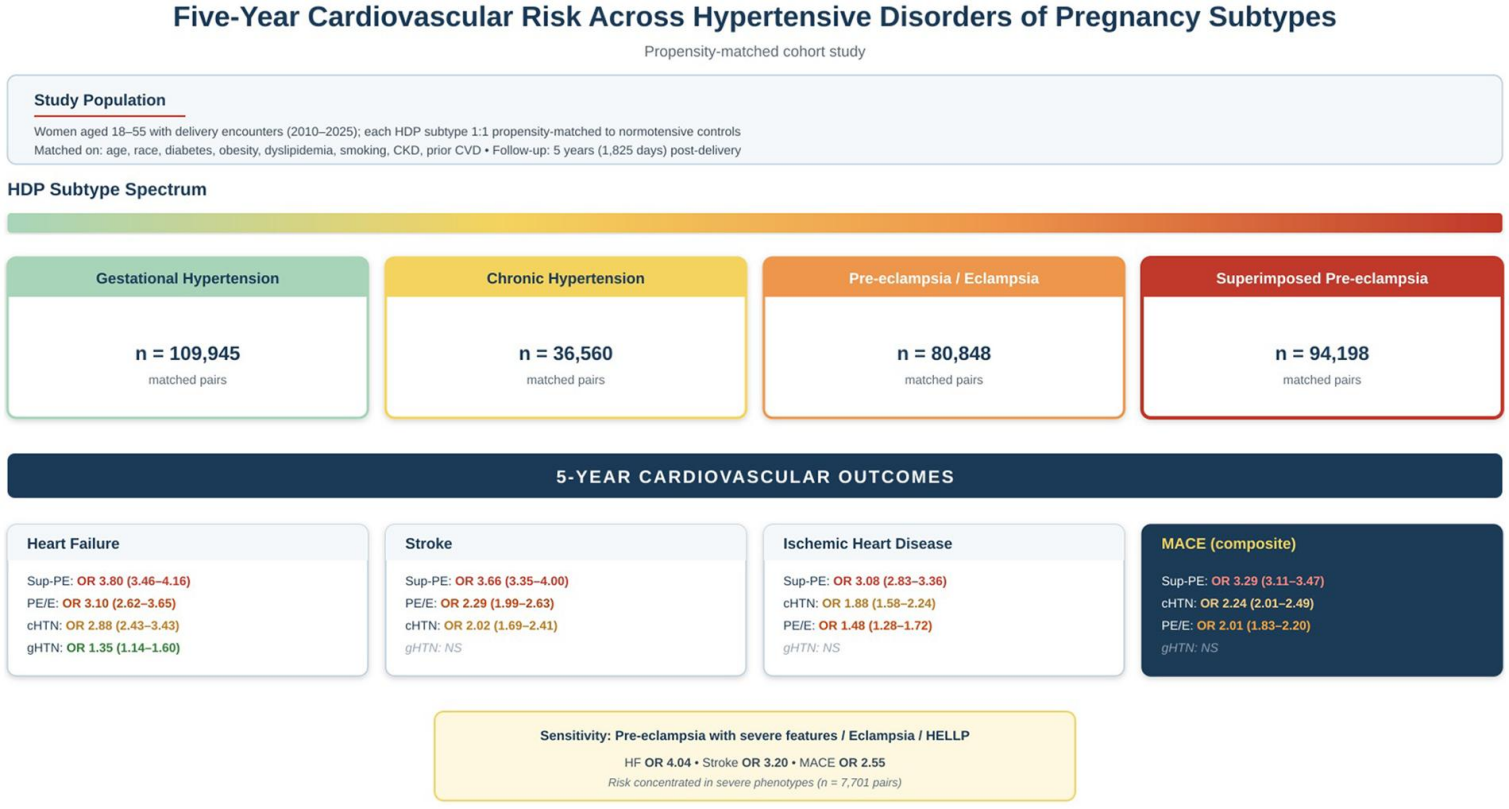
Five-Year Cardiovascular Risk Across Hypertensive Disorders of Pregnancy Subtypes: A Propensity-Matched Cohort Study. Central illustration summarizing five-year cardiovascular outcomes among women with hypertensive disorders of pregnancy (HDP) compared with propensity score–matched normotensive controls. The study population included women aged 18–55 years with delivery encounters between 2010 and 2025. HDP subtypes—gestational hypertension, chronic hypertension, preeclampsia/eclampsia, and superimposed preeclampsia—were defined using a hierarchical classification approach to ensure mutually exclusive groups. Each subtype was matched 1:1 to a separate normotensive control cohort. Adjusted odds ratios for heart failure, mortality, stroke, ischemic heart disease, and major adverse cardiovascular events (MACE) are shown. A sensitivity analysis restricted to preeclampsia with severe features, eclampsia, or HELLP syndrome demonstrated higher effect estimates for selected outcomes. Comparisons across HDP subtypes are indirect and based on comparison of effect estimates relative to matched controls. HDP, hypertensive disorders of pregnancy; gHTN, gestational hypertension; cHTN, chronic hypertension; PE/E, preeclampsia/eclampsia; Sup-PE, superimposed preeclampsia; HF, heart failure; IHD, ischemic heart disease; MACE, major adverse cardiovascular events; OR, odds ratio; CI, confidence interval; CKD, chronic kidney disease; CVD, cardiovascular disease; NS, not significant.

HDP comprise a heterogeneous group of conditions, including chronic hypertension, gestational hypertension, preeclampsia, and preeclampsia superimposed on chronic hypertension, with subtype definitions grounded in timing and clinical features as codified in obstetric and cardiovascular guidance (15–17). These disorders have distinct clinical phenotypes and likely differ in their pathophysiology and underlying baseline cardiometabolic risk (17). Consistent with this, a growing body of evidence indicates that cardiovascular risk appears to increase with disease severity and/or chronicity, with more severe phenotypes and those involving chronic hypertension generally showing higher subsequent risk (10, 11, 18). This dose-response relationship extends across multiple cardiovascular outcomes, including heart failure, stroke, and atrial fibrillation (3, 19–21). Among HDP subtypes, chronic hypertension with superimposed preeclampsia confers the highest stroke risk, followed by preeclampsia/eclampsia and gestational hypertension (18). Our findings are consistent with this pattern of differential risk across subtypes: superimposed preeclampsia demonstrated the largest odds ratios across endpoints, while preeclampsia/eclampsia and chronic hypertension also showed elevated risk, and gestational hypertension showed a narrower association profile in our data. These findings support conceptualizing HDP as a spectrum rather than a single entity, reflecting substantial heterogeneity in cardiovascular risk across subtypes (3, 17).

This pattern parallels our group's prior delivery-admission work showing graded acute cardiovascular maternal risk by subtype, with superimposed preeclampsia associated with the highest observed rates of maternal mortality, cerebrovascular events, and acute cardiac decompensation, followed by preeclampsia and chronic hypertension (22). In contrast, gestational hypertension demonstrated comparatively minimal excess risk relative to normotensive pregnancies (22). The present study extends these observations by demonstrating that this pattern of increasing risk persists well beyond the peripartum period, manifesting as markedly elevated five-year risks of heart failure, stroke, ischemic heart disease, and major adverse cardiovascular events (22).

The mechanisms linking HDP severity to long-term cardiovascular outcomes likely involve both shared predisposition (pre-pregnancy cardiometabolic risk) and pregnancy-associated vascular injury, with pregnancy acting as a physiologic “stress test” that unmasks or accelerates risk (9, 23, 24). Women with a history of preeclampsia demonstrate persistent vascular endothelial dysfunction, alterations in cardiac structure and function, and evidence of premature vascular aging and subclinical atherosclerosis years to decades after delivery (25, 26). Endothelial dysfunction may serve both as a shared risk factor predisposing to HDP and cardiovascular disease, and as a consequence of long-lasting metabolic and vascular injury caused by HDP itself (25, 27, 28). During pregnancy, placental malperfusion leads to increased secretion of anti-angiogenic factors, such as soluble fms-like tyrosine kinase-1 and soluble endoglin, which promote vascular inflammation, endothelial dysfunction, and maternal vascular injury (23). Whether this angiogenic imbalance persists long after delivery remains uncertain: some studies have reported higher postpartum soluble fms-like tyrosine kinase-1 levels and associations with postpartum hypertension and adverse cardiovascular structural changes, including increased carotid intima-media thickness and left ventricular posterior wall thickness, whereas others have shown normalization of angiogenic markers after pregnancy (29–32). Thus, angiogenic dysregulation may contribute to the vascular injury pathway linking HDP to later cardiovascular disease, but its role as a persistent postpartum biomarker requires further study. These findings are consistent with a broader syndrome of accelerated cardiovascular aging among women with prior HDP, supported by evidence of increased arterial stiffness, carotid intima-media thickness, and augmentation index persisting decades after pregnancy (24, 33). Although hypertension and other traditional cardiovascular risk factors account for approximately 64%–77% of the excess cardiovascular risk observed following HDP, substantial residual risk remains unexplained (34). Genetic epidemiology also supports shared inherited susceptibility between HDP (especially preeclampsia) and later cardiometabolic disease, consistent with a dual model of predisposition plus pregnancy-triggered injury (35).

Variation in cardiovascular risk among hypertensive disorder subtypes likely reflects differences in baseline cardiometabolic vulnerability, disease chronicity, and the magnitude of pregnancy-associated endothelial injury (36–38). Chronic hypertension exposes individuals to prolonged elevated blood pressure, myocardial, and vascular remodeling, which is a well-established driver of later heart failure, stroke, and atherosclerotic disease (36–41). The duration of hypertensive exposure is particularly important, as each additional year incrementally increases cardiovascular risk and exacerbates vascular and myocardial injury (42, 43). Acute endothelial injury from preeclampsia is associated with persistent vascular dysfunction, increased arterial stiffness, and lasting cardiac structural abnormalities that contribute to long-term cardiovascular morbidity (29, 44, 45). Even isolated preeclampsia, although transient during pregnancy, induces persistent vascular dysfunction and premature vascular aging, thereby contributing to long-term cardiovascular morbidity (23, 46, 47). When chronic hypertension is compounded by superimposed preeclampsia, the resulting “double hit” (chronic vascular disease plus acute pregnancy-related endothelial dysfunction and inflammation) is a plausible explanation for the consistently larger associations observed in this subgroup (23, 48, 49). Multiple recent cohort studies likewise identify superimposed preeclampsia as a particularly high-risk phenotype for early and later cardiovascular events, including stroke and heart failure (10, 11, 18).

Our subtype-specific five-year findings are supported by mechanistic evidence from our prior echocardiographic analysis, which demonstrated a graded spectrum of cardiac remodeling across HDP (50). In that study, echocardiographic parameters exhibited a pattern of increasing magnitude across subtypes, with the most pronounced abnormalities in superimposed preeclampsia, with less marked changes in other subtypes (50). Specifically, superimposed preeclampsia was associated with the greatest increases in left ventricular mass index and diastolic filling pressures, reflecting disproportionate structural remodeling and impaired relaxation. This prior work suggests that early cardiac remodeling may be an intermediate phenotype linking HDP subtype to downstream cardiovascular risk. The present analysis extends this trajectory by demonstrating a similar pattern in clinical cardiovascular events within five years postpartum, supporting a unified subtype spectrum model in which acute phenotype severity aligns with both subclinical cardiac changes and subsequent clinical outcomes (44, 45).

Prior studies have demonstrated increased long-term cardiovascular risk following both chronic hypertension and preeclampsia independently (4, 51, 52). Both chronic hypertension and preeclampsia/eclampsia were associated with elevated long-term cardiovascular risk, although the magnitude of risk was intermediate when compared with superimposed preeclampsia. This is broadly consistent with seminal meta-analytic evidence linking preeclampsia to later heart failure, coronary disease, and stroke, and with prospective cohort studies showing that HDP history is associated with a broad spectrum of cardiovascular conditions (4, 24). Differences in effect sizes across studies are expected given heterogeneity in exposure definitions (e.g., inclusion/exclusion of superimposed cases), outcome ascertainment, analytic approach (OR vs. HR), and follow-up windows (45). Together, these findings reinforce the importance of continued cardiovascular risk assessment and follow-up for women with chronic hypertension or preeclampsia/eclampsia after pregnancy (24, 53).

These findings were reinforced by a pre-specified sensitivity analysis restricted to women with severe pre-eclampsia, eclampsia, or HELLP syndrome. In this analysis, severe pre-eclampsia/eclampsia was associated with increased odds of multiple cardiovascular outcomes compared with matched normotensive controls. Effect estimates appeared higher than those in the primary pre-eclampsia/eclampsia cohort, although comparisons are descriptive and not based on formal statistical testing. This pattern aligns with prior evidence that cardiovascular risk scales with disease severity and supports the conclusion that much of the risk attributed to pre-eclampsia is concentrated in more severe phenotypes (44, 45). Practically, this supports incorporating severity descriptors (preeclampsia with severe features, eclampsia, HELLP), rather than preeclampsia diagnosis alone, into postpartum cardiovascular risk stratification, and highlights the need for a shared clinical lexicon between obstetrics and cardiology to ensure accurate communication of risk.

Gestational hypertension demonstrated a relatively limited association with long-term cardiovascular outcomes compared with other hypertensive disorder subtypes, with an increased risk observed primarily for heart failure. This finding should be interpreted in the context of mixed prior literature: meta-analyses suggest gestational hypertension confers modest long-term risk for cardiovascular disease and heart failure, with more variable associations for stroke depending on populations, follow-up, and adjustment (56). A key possibility is that gestational hypertension represents a heterogeneous group, including individuals with transient pregnancy-limited hypertension and others with unrecognized predisposition to chronic hypertension. Prior studies evaluating long-term cardiovascular outcomes following gestational hypertension have reported mixed findings, with some demonstrating modest increases in cardiovascular risk and others showing attenuation after adjustment for baseline risk factors (51, 52, 54). Importantly, the absence of broad cardiovascular associations in this subgroup should not be interpreted as reassurance, as gestational hypertension may still identify individuals who could benefit from individualized postpartum cardiovascular risk assessment (24, 53).

The *post hoc* analysis demonstrated a graded increase in the composite of postpartum essential hypertension diagnosis or antihypertensive treatment after gestational hypertension, preeclampsia/eclampsia, and severe preeclampsia/eclampsia. This pattern supports the importance of early and continued blood-pressure assessment after these pregnancy phenotypes and suggests that development of postpartum hypertension may contribute to their subsequent cardiovascular risk. Interpretation should account for the operational definition: antihypertensive medication use was included to improve capture across the electronic health record network, but some included agents may be prescribed for indications other than hypertension.

These findings have practical implications for how HDP history is carried forward after delivery. Existing ACOG and SMFM recommendations already emphasize postpartum blood-pressure assessment, structured discharge planning, and transition to ongoing care after HDP, and AHA statements recognize adverse pregnancy outcomes as opportunities for CVD prevention (3, 55, 56). Our results suggest that this history should not be treated simply as present or absent. The largest and most consistent five-year associations were seen after superimposed preeclampsia, consistent with Hu et al. and Kwak et al., who also identified superimposed preeclampsia as a particularly high-risk subtype for later cardiovascular events (10, 11). Chronic hypertension and preeclampsia/eclampsia also carried elevated risk across several outcomes, whereas gestational hypertension showed a narrower profile but remained associated with subsequent hypertension. In practice, this supports documenting HDP subtype and severity during postpartum transition of care and using that information to guide the intensity of follow-up. Women with superimposed preeclampsia, chronic hypertension, or preeclampsia/eclampsia may benefit from more deliberate linkage to primary care or cardiology, earlier assessment of blood-pressure control, and longitudinal management of hypertension, obesity, dyslipidemia, and diabetes (24, 57). For gestational hypertension, the findings support postpartum blood-pressure reassessment and monitoring for progression to chronic hypertension, with follow-up intensity guided by additional cardiometabolic risk factors. Although our data support earlier risk recognition and prioritization by HDP phenotype, they do not establish specific visit intervals, testing strategies, or referral thresholds.

### 4.1 Limitations

Several limitations should be acknowledged. First, this was an observational analysis using real-world electronic health record data, and residual confounding cannot be fully excluded despite careful propensity score matching. Factors such as socioeconomic status, health behaviors, access to care, and medication adherence were not consistently available and may have influenced long-term cardiovascular outcomes. Because each hypertensive disorder subtype was matched to an independent control cohort, comparisons across subtypes are indirect and based on visual comparison of effect estimates rather than formal statistical testing. Although most standardized mean differences were below accepted thresholds, small residual imbalances in certain baseline variables (e.g., heart failure in the superimposed preeclampsia cohort) persisted and may have influenced effect estimates.

Second, hypertensive disorder subtypes and cardiovascular outcomes were identified using administrative diagnostic codes, which may introduce misclassification and may not fully capture disease severity or timing. Additionally, diagnostic criteria for preeclampsia evolved during the study period, particularly following updated guideline definitions in 2014 (13). Proteinuria was no longer required for diagnosis when hypertension was accompanied by other end-organ features, such as thrombocytopenia, renal insufficiency, impaired liver function, pulmonary edema, or neurologic symptoms. As a result, the preeclampsia/eclampsia cohort may include a heterogeneous mixture of proteinuric and nonproteinuric phenotypes, as well as patients with differing degrees and types of end-organ involvement. Because long-term cardiovascular risk may vary according to the specific clinical phenotype of preeclampsia, this change in diagnostic criteria could have influenced case ascertainment and may have attenuated or modified observed associations. Our sensitivity analysis restricted to severe preeclampsia, eclampsia, and HELLP syndrome partially addresses disease severity, but diagnostic codes do not allow reliable separation of proteinuric from nonproteinuric disease or assessment of the specific end-organ criteria used for diagnosis. Additionally, information on fetal growth restriction, preterm birth, and other clinical markers of placental dysfunction was not separately extracted or included in the matching model. These adverse pregnancy outcomes may identify a greater burden of placental disease and have themselves been associated with subsequent maternal cardiovascular risk (57, 58). Their absence may therefore contribute to residual confounding and limits our ability to determine whether the observed subtype-specific associations are independent of coexisting placental dysfunction. Future studies integrating HDP subtype with gestational age at delivery, fetal growth, and other placental phenotypes may provide more refined postpartum cardiovascular risk stratification.

Third, detailed information on blood pressure trajectories, medication use, and postpartum risk factor management was limited, precluding adjustment for changes in treatment or preventive care after pregnancy that may have modified cardiovascular risk. Fourth, the study population was relatively young, and absolute event rates were low over the five-year follow-up period, limiting assessment of longer-term outcomes and reducing precision for less frequent events; mortality findings in particular should be interpreted cautiously. Fifth, data were derived from a large, multi-institutional network with variability in documentation practices and follow-up patterns, and outcomes occurring outside participating health systems may not have been captured, leading to potential under-ascertainment.

Finally, given the observational design, causal inferences cannot be made, and findings should be viewed as hypothesis-generating. Although the sensitivity analysis isolates preeclampsia with severe features and eclampsia, severity ascertainment still relies on administrative coding and may under-capture clinically severe disease that lacked a formal severe-features designation at the time of delivery.

## 5 Conclusion

Long-term cardiovascular risk following HDP varies substantially by subtype, with larger effect estimates observed among women with superimposed preeclampsia. Women with chronic hypertension and preeclampsia/eclampsia also demonstrated elevated risk for multiple adverse cardiovascular outcomes compared with normotensive pregnancies, whereas gestational hypertension was associated with a more limited cardiovascular risk profile. These findings demonstrate the heterogeneity of HDP and suggest that consideration of disease subtype may be relevant when evaluating long-term cardiovascular risk after pregnancy. Further prospective studies are needed to clarify absolute risk, underlying mechanisms, and the most effective approaches to postpartum cardiovascular surveillance and prevention.

## Data Availability

The datasets presented in this article are not readily available because the data used in this study are derived from the TriNetX Research Network. Access to the data is subject to institutional agreements and cannot be publicly shared. Requests to access the datasets should be directed to https://trinetx.com/data-sets-analytics/.
