# Supplementary material for: Five-year cardiovascular outcomes across hypertensive disorders of pregnancy subtypes: a propensity-matched analysis
Source: Front Cardiovasc Med. 2026 Jul 24;13:1875409. doi: 10.3389/fcvm.2026.1875409 (PMC13447380; doi:10.3389/fcvm.2026.1875409)
Supplement: Supplementary file 1 [file Table1.docx]

**Supplemental:**

Supplemental Table S1. Delivery Eligibility and Control Cohort Exclusion Criteria

| **Status** | **Criterion** | **Codes** | **Operational definition** |
| --- | --- | --- | --- |
| Required | Delivery encounter | ICD-10-CM O80-O82; O75.82 | Encounter for delivery or specified labor with planned cesarean delivery. |
| Required | Delivery procedure | ICD-10-PCS 10D00Z0, 10D00Z1, 10D00Z2, 10D07Z3-10D07Z8, 10E0XZZ | Extraction or delivery of products of conception. |
| Excluded | Ectopic or molar pregnancy | ICD-10-CM O00, O01 | Ectopic pregnancy or hydatidiform mole. |
| Excluded | Pregnancy loss or termination-related diagnosis | ICD-10-CM O03, O04, O07, O08 | Spontaneous abortion; complications following induced termination; failed attempted termination; or complications following ectopic or molar pregnancy. |
| Excluded | Abortion procedure | ICD-10-PCS 10A0 | Abortion of products of conception. |
| Excluded from control | Hypertensive disorder of pregnancy | ICD-10-CM O10-O16, including O14.2; ICD-9-CM 642.2, 642.3, 642.4, 642.7 | Pre-existing or gestational hypertension, preeclampsia/eclampsia, HELLP syndrome, superimposed preeclampsia, edema/proteinuria without hypertension, and unspecified maternal hypertension. |

Abbreviations: HDP, hypertensive disorders of pregnancy; HELLP, hemolysis, elevated liver enzymes, and low platelet count; ICD-9-CM, International Classification of Diseases, Ninth Revision, Clinical Modification; ICD-10-CM, International Classification of Diseases, Tenth Revision, Clinical Modification; ICD-10-PCS, International Classification of Diseases, Tenth Revision, Procedure Coding System.

**Supplemental Table S2. Baseline Characteristics After Propensity Score Matching**

Gestational Hypertension (Cohort 1) vs Control (Cohort 2)

| Characteristic | Gestational HTN (N=109,945) | Control (N=109,945) | P-value | Std. Diff |
| --- | --- | --- | --- | --- |
| Age at index, years (mean ± SD) | 29.2 ± 6.0 | 29.2 ± 6.0 | 0.931 | <0.001 |
| White, n (%) | 67,635 (61.5) | 67,669 (61.5) | 0.882 | 0.001 |
| Not Hispanic or Latino, n (%) | 75,078 (68.3) | 75,139 (68.3) | 0.780 | 0.001 |
| Hispanic or Latino, n (%) | 16,122 (14.7) | 16,103 (14.6) | 0.909 | <0.001 |
| Black or African American, n (%) | 20,433 (18.6) | 20,386 (18.5) | 0.797 | 0.001 |
| Asian, n (%) | 4,399 (4.0) | 4,424 (4.0) | 0.786 | 0.001 |
| Chronic kidney disease | 189 (0.2) | 162 (0.1) | 0.149 | 0.006 |
| Overweight/obesity | 25,603 (23.3) | 25,645 (23.3) | 0.832 | 0.001 |
| Dyslipidemia | 2,845 (2.6) | 2,828 (2.6) | 0.819 | 0.001 |
| Diabetes mellitus | 2,853 (2.6) | 2,832 (2.6) | 0.778 | 0.001 |
| Nicotine dependence | 8,570 (7.8) | 8,589 (7.8) | 0.880 | 0.001 |
| Atrial fibrillation/flutter | 157 (0.1) | 143 (0.1) | 0.419 | 0.003 |
| Ischemic heart disease | 356 (0.3) | 318 (0.3) | 0.143 | 0.006 |
| Heart failure | 157 (0.1) | 121 (0.1) | 0.031 | 0.009 |
| Asthma | 12,141 (11.0) | 12,123 (11.0) | 0.902 | 0.001 |
| Polycystic ovarian syndrome | 3,724 (3.4) | 3,697 (3.4) | 0.750 | 0.001 |

**Supplemental Table S3: Baseline Characteristics After Propensity Score Matching**

Chronic Hypertension (Cohort 1) vs Control (Cohort 2)

| Characteristic | Chronic HTN (N=36,560) | Control (N=36,560) | P-value | Std. Diff |
| --- | --- | --- | --- | --- |
| Age at index, years (mean ± SD) | 31.0 ± 6.0 | 31.0 ± 6.0 | 0.800 | 0.002 |
| White, n (%) | 18,600 (50.9) | 18,623 (50.9) | 0.865 | 0.001 |
| Not Hispanic or Latino, n (%) | 23,604 (64.6) | 23,639 (64.7) | 0.787 | 0.002 |
| Hispanic or Latino, n (%) | 5,235 (14.3) | 5,241 (14.3) | 0.950 | <0.001 |
| Black or African American, n (%) | 9,252 (25.3) | 9,252 (25.3) | 1.000 | <0.001 |
| Asian, n (%) | 1,690 (4.6) | 1,685 (4.6) | 0.930 | 0.001 |
| Chronic kidney disease | 228 (0.6) | 196 (0.5) | 0.119 | 0.012 |
| Overweight/obesity | 9,719 (26.6) | 9,796 (26.8) | 0.520 | 0.005 |
| Dyslipidemia | 1,055 (2.9) | 1,108 (3.0) | 0.247 | 0.009 |
| Diabetes mellitus | 2,173 (5.9) | 2,205 (6.0) | 0.618 | 0.004 |
| Nicotine dependence | 3,033 (8.3) | 3,045 (8.3) | 0.872 | 0.001 |
| Atrial fibrillation/flutter | 121 (0.3) | 126 (0.3) | 0.750 | 0.002 |
| Ischemic heart disease | 291 (0.8) | 247 (0.7) | 0.057 | 0.014 |
| Heart failure | 295 (0.8) | 236 (0.6) | 0.010 | 0.019 |
| Asthma | 3,986 (10.9) | 3,982 (10.9) | 0.962 | <0.001 |
| Polycystic ovarian syndrome | 1,128 (3.1) | 1,173 (3.2) | 0.340 | 0.007 |

**Supplemental Table S4. Baseline Characteristics After Propensity Score Matching**

Superimposed Pre-eclampsia (Cohort 1) vs Control (Cohort 2)

| Characteristic | Superimposed Pre-eclampsia (N=94,198) | Control (N=94,198) | P-value | Std. Diff |
| --- | --- | --- | --- | --- |
| Age at index, years (mean ± SD) | 32.3 ± 6.4 | 32.2 ± 6.3 | <0.001 | 0.018 |
| White, n (%) | 48,210 (51.2) | 48,499 (51.5) | 0.183 | 0.006 |
| Not Hispanic or Latino, n (%) | 65,278 (69.3) | 65,417 (69.4) | 0.487 | 0.003 |
| Hispanic or Latino, n (%) | 12,080 (12.8) | 12,323 (13.1) | 0.095 | 0.008 |
| Black or African American, n (%) | 28,678 (30.4) | 28,591 (30.4) | 0.663 | 0.002 |
| Asian, n (%) | 3,588 (3.8) | 3,581 (3.8) | 0.933 | <0.001 |
| Chronic kidney disease | 1,391 (1.5) | 1,166 (1.2) | <0.001 | 0.021 |
| Overweight/obesity | 35,434 (37.6) | 36,092 (38.3) | 0.002 | 0.014 |
| Dyslipidemia | 6,543 (6.9) | 6,645 (7.1) | 0.357 | 0.004 |
| Diabetes mellitus | 8,668 (9.2) | 8,856 (9.4) | 0.136 | 0.007 |
| Nicotine dependence | 10,846 (11.5) | 11,299 (12.0) | 0.001 | 0.015 |
| Atrial fibrillation/flutter | 408 (0.4) | 405 (0.4) | 0.916 | <0.001 |
| Ischemic heart disease | 1,082 (1.1) | 1,068 (1.1) | 0.761 | 0.001 |
| Heart failure | 1,011 (1.1) | 774 (0.8) | <0.001 | 0.026 |
| Asthma | 14,461 (15.4) | 14,930 (15.8) | 0.003 | 0.014 |
| Polycystic ovarian syndrome | 5,093 (5.4) | 5,594 (5.9) | <0.001 | 0.023 |

**Supplemental Table S5. Baseline Characteristics After Propensity Score Matching**

Pre-eclampsia / Eclampsia (Cohort 1) vs Control (Cohort 2)

| Characteristic | Pre-eclampsia/Eclampsia (N=80,848) | Control (N=80,848) | P-value | Std. Diff |
| --- | --- | --- | --- | --- |
| Age at index, years (mean ± SD) | 29.1 ± 6.4 | 29.1 ± 6.4 | 0.899 | 0.001 |
| White, n (%) | 41,601 (51.5) | 41,672 (51.5) | 0.724 | 0.002 |
| Not Hispanic or Latino, n (%) | 46,433 (57.4) | 46,472 (57.5) | 0.844 | 0.001 |
| Hispanic or Latino, n (%) | 17,131 (21.2) | 17,157 (21.2) | 0.874 | 0.001 |
| Black or African American, n (%) | 16,377 (20.3) | 16,364 (20.2) | 0.936 | <0.001 |
| Asian, n (%) | 4,014 (5.0) | 4,000 (4.9) | 0.873 | 0.001 |
| Chronic kidney disease | 227 (0.3) | 208 (0.3) | 0.362 | 0.005 |
| Overweight/obesity | 14,423 (17.8) | 14,461 (17.9) | 0.805 | 0.001 |
| Dyslipidemia | 1,780 (2.2) | 1,762 (2.2) | 0.760 | 0.002 |
| Diabetes mellitus | 3,167 (3.9) | 3,183 (3.9) | 0.838 | 0.001 |
| Nicotine dependence | 5,024 (6.2) | 5,017 (6.2) | 0.942 | <0.001 |
| Atrial fibrillation/flutter | 109 (0.1) | 93 (0.1) | 0.260 | 0.006 |
| Ischemic heart disease | 281 (0.3) | 264 (0.3) | 0.466 | 0.004 |
| Heart failure | 213 (0.3) | 166 (0.2) | 0.016 | 0.012 |
| Asthma | 7,119 (8.8) | 7,143 (8.8) | 0.833 | 0.001 |
| Polycystic ovarian syndrome | 1,962 (2.4) | 2,018 (2.5) | 0.369 | 0.004 |

**Supplemental Table S6. Baseline Characteristics After Propensity Score Matching**

Severe Pre-eclampsia, Eclampsia, or HELLP (Cohort 1) vs Control (Cohort 2)

| **Characteristic** | **Severe PE / Eclampsia (N=7,701)** | **Control (N=7,701)** | **P-value** | **Std. Diff** |
| --- | --- | --- | --- | --- |
| Age at index, years (mean ± SD) | 29.1 ± 6.5 | 29.1 ± 6.5 | 0.994 | <0.001 |
| White, n (%) | 3,690 (47.9) | 3,693 (48.0) | 0.961 | 0.001 |
| Black or African American, n (%) | 1,809 (23.5) | 1,807 (23.5) | 0.970 | 0.001 |
| Hispanic or Latino, n (%) | 1,194 (15.5) | 1,199 (15.6) | 0.911 | 0.002 |
| Asian, n (%) | 342 (4.4) | 343 (4.5) | 0.969 | 0.001 |
| Not Hispanic or Latino, n (%) | 4,333 (56.3) | 4,345 (56.4) | 0.845 | 0.003 |
| Overweight / obesity, n (%) | 1,249 (16.2) | 1,253 (16.3) | 0.930 | 0.001 |
| Diabetes mellitus, n (%) | 334 (4.3) | 351 (4.6) | 0.506 | 0.011 |
| Dyslipidemia, n (%) | 142 (1.8) | 147 (1.9) | 0.767 | 0.005 |
| Nicotine dependence, n (%) | 306 (4.0) | 305 (4.0) | 0.967 | 0.001 |
| Asthma, n (%) | 662 (8.6) | 667 (8.7) | 0.886 | 0.002 |
| Polycystic ovary syndrome, n (%) | 157 (2.0) | 166 (2.2) | 0.613 | 0.008 |
| Chronic kidney disease, n (%) | 33 (0.4) | 26 (0.3) | 0.361 | 0.015 |
| Heart failure, n (%) | 38 (0.5) | 33 (0.4) | 0.552 | 0.010 |
| Ischemic heart disease, n (%) | 37 (0.5) | 41 (0.5) | 0.650 | 0.007 |
| Atrial fibrillation / flutter, n (%) | 13 (0.2) | 11 (0.1) | 0.683 | 0.007 |
| Cesarean section, n (%) | 15 (0.2) | 14 (0.2) | 0.853 | 0.003 |

**Supplemental Table S7. Five-Year Outcomes; Severe Pre-eclampsia / Eclampsia vs. Normotensive Control**

| **Outcome** | **Severe PE/E n (%) vs control** | **OR (95% CI) — sensitivity** |
| --- | --- | --- |
| Heart failure | 88 (1.14) vs 22 (0.29) | 4.04 (2.53–6.44) |
| Stroke | 114 (1.48) vs 36 (0.47) | 3.20 (2.20–4.66) |
| MACE (composite) | 198 (2.57) vs 79 (1.03) | 2.55 (1.96–3.31) |
| Pulmonary embolism | 44 (0.57) vs 24 (0.31) | 1.84 (1.12–3.03) |
| Ischemic heart disease | 47 (0.61) vs 28 (0.36) | 1.68 (1.05–2.69) |
| New onset hypertension or antihypertensive treatment | 3,301/7,701 (42.863%) vs 613/7,701 (7.954%) | 8.68 (7.62–9.89) |
